# Interleukin-18 in Brazilian Rheumatoid Arthritis Patients: Can Leflunomide Reduce It?

**DOI:** 10.1155/2021/6672987

**Published:** 2021-05-10

**Authors:** Pablo Ramon Gualberto Cardoso, Claudia Diniz Lopes Marques, Kamila de Melo Vilar, Andrea Tavares Dantas, Angela Luzia Branco Pinto Duarte, Ivan da Rocha Pitta, Maira Galdino da Rocha Pitta, Moacyr Jesus Barreto de Melo Rêgo

**Affiliations:** ^1^Laboratory of Immunomodulation and New Therapeutic Approaches (LINAT), Research Group on Immunomodulation and New Therapeutic Approaches Suely Galdino (Nupit SG), Federal University of Pernambuco, Recife, Brazil; ^2^Clinics Hospital, Federal University of Pernambuco, Recife, Brazil

## Abstract

**Objectives:**

Rheumatoid arthritis affects about 1% of the world's population. This is a chronic autoimmune disease. It is predominant in females with progressive joint damage. Immune cells are involved, especially Th1/Th17 lymphocytes and their inflammatory cytokines. These proteins have different functions in the immune system, such as IL-16 is a chemotactic factor; IL-18 can activate NF*κ*B transcription producing inflammatory proteins; IL-31 can activate the JAK/STAT pathway which leads to the production of inflammatory factors in chronic diseases; IL-33 promotes IL-16 secretion which causes lymphocyte recruitment, and IL-32 and IL-34 appear to increase TNF secretion by macrophages activation in AR. The aim of this study was to evaluate serum levels of IL-16, IL-18, IL-31, IL-32, IL-33, and IL-34 and compare them with the severity and treatment of RA patients if there are any correlations.

**Methods:**

A total of 140 RA patients and 40 healthy donors were recruited from the Department of Rheumatology at Hospital das Clínicas from the Federal University of Pernambuco. 60 AR patients were naïve for any treatment. Serum cytokine levels were determined using an ELISA kit.

**Results:**

Serum IL-16 (*p* = 0.0491), IL-18 (*p* < 0.0001), IL-31 (*p* = 0.0004), and IL-32 (*p* = 0.0040) levels were significantly increased in RA patients compared with healthy donors. It was observed that patients using leflunomide had the lowest IL-18 levels, close to controls levels (*p* = 0.0064).

**Conclusion:**

IL-16, IL-18, IL-31, and IL-32 are increased in the serum of RA patients. IL-18 is at lower levels in those AR who are taking leflunomide as treatment.

## 1. Introduction

Rheumatoid arthritis (RA) is a chronic inflammatory disease. This disease mainly affects women. It is estimated that 1% of the world's population has it. RA is associated with progressive and irreversible joint damage, causing a reduced quality of life and increased mortality [1]. In RA patients, there is an imbalance between anti-inflammatory and proinflammatory cytokines and Th1/Th17 cell responses have been implicated [2]. The key proinflammatory cytokines involved in RA include TNF, IFN-*γ*, IL-1*β*, IL-6, IL-8, and IL-23/IL-17 [3]. Little is known regarding the role of other cytokines, such as IL-16, IL-18, IL-31, IL-32, IL-33, and IL-34, in the pathophysiology of RA. Thus, we will focus our efforts to better understand the role of these other cytokines.

IL-16 is produced by CD4+ T cells [4, 5] and promotes immune cell recruitment by the CD40 ligand [6]. IL-17 A and IL-18 induce IL-16 production [7, 8], which may be associated with the progression of RA as it is directly correlated with metalloproteinase-3 production in the RA synovium [9].

IL-18 and IL-33 are members of the interleukin-1 (IL-1) family. IL-18 is involved in the host defense response by recruiting cells into target tissues, initiating an inflammatory response. This cytokine promotes the production of granulocyte-macrophage colony-stimulating factor and nitric oxide [10–12] and leads the pathway to NF*κ*B transcription [13]. NF*κ*B activation induces IL-1*β* production [14]. Furthermore, IL-18 induces IL-16 [8] and other inflammatory cytokines involved in RA development and progression [15]. IL-33 activates NF*κ*B and MAP kinases and also stimulates the production of Th2-associated cytokines in vitro [16]. In inflammatory airway diseases, IL-33 is associated with eosinophil recruitment [17]. Moreover, IL-33 is positively correlated with the generation of autoantibodies in RA [18].

Recent studies have demonstrated that IL-31 plays an important role in the induction of chronic inflammation [19, 20] and regulates tissue remodeling [21]. It belongs to the IL-6 family, a potential inflammatory cytokine [22, 23]. Another proinflammatory cytokine is IL-32, which may be a multifunctional protein. It acts as an endogenous regulator of cytokine production and can activate macrophages to secrete TNF [24].

IL-34 also activates macrophages as well as monocytes via binding to the colony-stimulating factor-1 receptor (CSF-1R), promoting the proliferation, survival, and differentiation of phagocytes, such as macrophages, osteoclasts, and Langerhans cells [25, 26].

The objective of our study was to evaluate the serum profile of IL-16, IL-18, IL-31, IL-32, IL-33, and IL-34 in patients with RA and to correlate these data with other laboratory and clinical data.

## 2. Materials and Methods

### 2.1. Study Population

Serum samples were obtained from 180 volunteers by venipuncture. A total of 140 patients with RA (130 women, 10 men; mean age 54.0 (±12.0) years) were recruited from the Department of Rheumatology at the Hospital of Clinics at the Federal University of Pernambuco (UFPE). Demographics, current medications, and clinical and laboratory data were recorded (Table 1). Receiving biological treatment was considered an exclusion criterion. Among the recruited patients, 60 were naive for treatment. The diagnosis of RA was established by ACR/EULAR diagnostic criteria [27], and patients were invited to participate in the study via outpatient care. Forty healthy donors matched by age and sex (51.5 ± 8.5 years) were included in the control group (CG), and all of them were free of any autoimmune or inflammatory disorders. The study was approved by the ethics committee of the UFPE (protocols no. 53555116.0.0000.5208), according to the principles of the Declaration of Helsinki. All subjects provided written consent to participate.

### 2.2. Clinical Data

Individual disease activity was quantified using the Clinical Disease Activity Index (CDAI) [27] and Disease Activity Score for 28 joints (DAS28) [28]. Laboratory data from RA patients such as erythrocyte sedimentation rate (ESR) and rheumatoid factor (RF) positivity were recorded at HC-UFPE.

### 2.3. Cytokine Measurement

Serum cytokine levels for IL-16, IL-18, IL-31, IL-32, IL-33, and IL-34 were determined in duplicate using an ELISA kit, according to the manufacturer's instructions (R&D Systems, Minneapolis, MN, USA) at our research center Laboratory of Immunomodulation and New Therapeutic Approaches (LINAT). The lower detection limits were as follows: 7.81 pg/ml for IL-16; 46.80 pg/ml for IL-18; 62.5 pg/ml for IL-31; 39.06 pg/ml for IL-32; 11.71 pg/ml for IL-33; and 31.25 pg/ml for IL-34.

### 2.4. Statistical Analysis

Statistical analyses of the data were performed using the GraphPad Prism 6.1 (GraphPad Software Inc., San Diego, CA) statistical program. Fisher's and chi-square tests were used to validate the gender normality, and the Shapiro–Wilk normality test verified normality of the age data. Numerical data were expressed as the mean ± standard error (SE) (age and cytokine measurements) or the median (maximum/minimum) (ESH) if they were in a normal distribution or as the median and interquartile range (IQR) if they were not. The Mann–Whitney test was used to compare serum cytokine levels. Pearson's and Spearman's correlation coefficients were used for correlation analyses. Data were evaluated as weak (<0.3), moderate (0.3–0.7), or strong (>0.7). A probability value of *p* < 0.05 was considered statistically significant.

## 3. Results

The study included 140 patients, with an average age of 54.0 (±12.0) years and a mean disease duration of 10.0 (±7.8) years. The CG consisted of 40 volunteers with an average age of 51.5 (±8.5) years. The RA and CGs were recruited from the Rheumatology Division at the Hospital of the Clinics at the Federal University of Pernambuco. RA patients were included after fulfilling at least four ACR/EULAR (2010) [27] classification criteria for RA. Table 1 presents the clinical data. Individuals in the healthy CG were lacking clinical symptoms associated with systemic inflammation, and family histories were free from rheumatic diseases as well. Altogether, the groups were homogeneous for sex and age.

### 3.1. Serum Cytokine Levels in RA Patients and Controls

**Figure 1:**
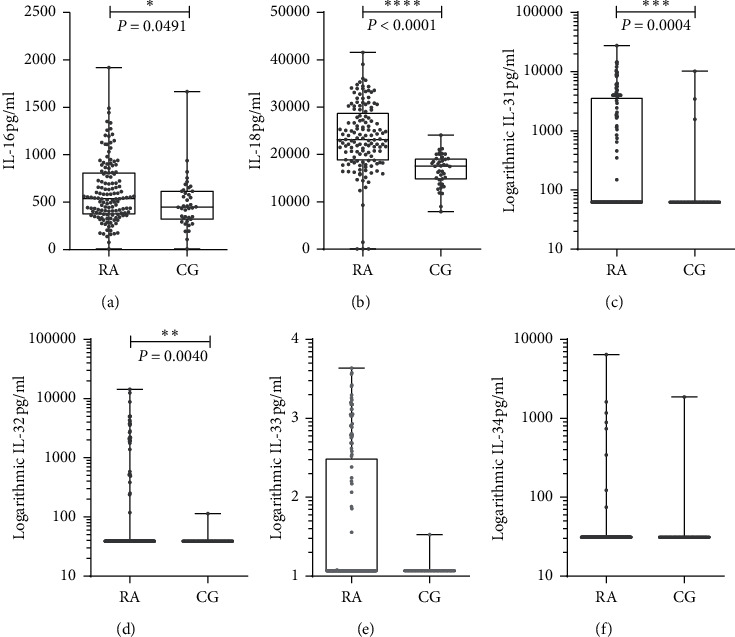
Cytokines serum evaluation: IL-16 (a), IL-18 (b), IL-31 (c), IL-32 (d), IL-33 (e), and IL-34 (f) levels in RA patients (140) and control group [29]. IL-16, -18, - 31, and -32 were found at high levels in RA patients (Mann–Whitney test).

Most of the cytokine levels were higher in RA patients than in the CG. The most prominent difference was observed for IL-18 expressed by the median (max-min) *p* value in the AR [23150.0 (41548.0–46.8, *p* < 0.0001] and CG [17562.0 (24084.0–7944.0)], followed by IL-16 [539.1 (1919.1–7.8) *p* = 0.0491] and [449.4 (1664.6–7.8)], IL-31 [62.5 (27550.0–62.5) *p* = 0.0004] and [62.5 (10194.3–62.5)], and IL-32 [39.0 (14425.3–39.0) *p* < 0.0040] and [39.0 (115.0–39.0), all levels that were significantly higher in RA patients compared with controls (Figure 1). There was a trend toward upregulated serum levels for IL-33 and IL-34 in RA patients, but these data were not statistically significant.

### 3.2. Association of Serum Interleukin Levels and Drug Treatment

**Figure 2:**
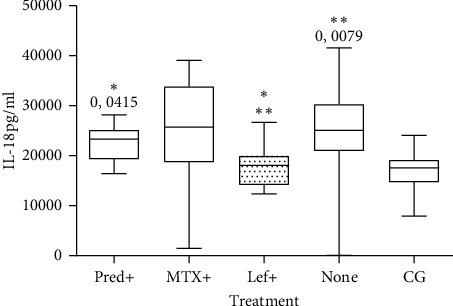
Cytokine levels of patients being treated or not. RA patients who underwent leflunomide treatment (Lef + *n* = 7) exhibited lower serum IL-18 levels than patients who did not undergo any treatment (*n* = 60) (*p* = 0.0079) as well as those who were treated with prednisone (Pred + *n* = 10) (*p* = 0.0415). There was no statistical difference with the CG (Mann–Whitney test). MTX+ = methotrexate treatment *n* = 12.

We analyzed cytokine levels to see if there was any relationship with clinical parameters using the Pearson and Spearman correlation. However, no statistically significant differences were found with CDAI or DAS28 criteria. In our study, cytokine levels were also evaluated in patients who were only being treated with a single drug (leflunomide, prednisone, or methotrexate). Only IL-18 levels showed a significant difference with treatment. Additionally, IL-18 levels in the untreated RA group and healthy controls were also compared. It was observed that IL-18 levels in patients undergoing leflunomide treatment were the lowest compared to the healthy control group, and these were significantly different than those of patients who were not undergoing treatment (*p* = 0.0079) or those who were being treated with prednisone (*p* = 0.0415) (Figure 2). IL-18 levels in prednisone-treated patients were similar to those in the control group. We did not find any association between the levels of other cytokines and treatment.

## 4. Discussion

Rheumatoid arthritis is a common autoimmune disease that affects mainly women. Inflammatory and anti-inflammatory cytokines might be involved in worsening or enhancing the progression of this condition and may serve as biomarkers for early diagnosis. Also, our group has already demonstrated the importance of some cytokines, such as IL-17A, and IL-22, in the Brazilian RA population [30–32]. This study evaluated the levels of IL-16, IL-18, IL-31, IL-32, IL-33, and IL-34 in RA, demonstrating that IL-16, IL-18, IL-31, and IL-32 were upregulated in RA patients compared to age- and sex-matched healthy donors. The results of this study showed upregulated IL-16 levels, which corroborates previous studies on RA patients [33] However, unlike Murota et al.) [9], we found no clinical association between RA progression and IL-16 levels.

IL-18 has been associated with the severity of RA in the synovium, acting with IL-15 and IL-12 [10] or myeloperoxidase to increase damage [34]. In addition, it has been found that IL-18 is upregulated in systemic lupus erythematosus (SLE) serum [35] and gout [36] and may play an important role in proinflammatory cytokine production. IL-18 upregulates the expression of membrane-bound RANKL, soluble RANKL, M-CSF, GM-CSF, and osteoprotegerin (OPG) in cultured fibroblasts. IL-18 inducing effects may promote the maturation of osteoclasts leading to bone degradation in RA [37]. IL-18 has also been identified as a cytokine produced in viral defense and is produced by monocytes by activating inflammasomes [38]. Previous studies from our group showed that RA PBMCs stimulated with anti-CD3 and anti-CD28 express higher levels of IL-18 in culture supernatants [39]. With these findings, we questioned whether circulating lymphocytes are IL-18 secretors in RA, as there are high levels of this interleukin in RA serum. This is the first study examining serum IL-18 levels in Brazilian RA patients.

IL-31 belongs to the gp130/IL-6 cytokine family. It is expressed preferentially by activated Th2 CD4+ T cells, and its pleiotropic effects on the immune system and RA remains unclear. During inflammation in the respiratory system, IL-31 appears to induce Th1/Th17 differentiation and it acts as a stimulus for the production of IL-4 and IL-5. Its role in chronic type 2 inflammation, such as fibrosis and tissue remodeling, is still unknown [23,40]. Our data show that RA patients exhibit high serum levels of IL-31. However, no clinical correlations or associations were identified. Thus, further work is necessary for understanding its physiological function in this pathological condition.

Leflunomide is an oral disease-modifying antirheumatic drug (DMARD) used for RA. This drug improves the patients' clinical condition and, thus, their quality of life [29]. Leflunomide downregulates the rates of MMP-1, MMP-3, IL-6, and IL-10, suggesting its action in the immunopathology of RA [41]. Our findings showed that patients undergoing treatment with leflunomide exhibited lower IL-18 levels than patients undergoing other types of treatment, such as methotrexate or prednisone. In RA patients undergoing leflunomide treatment, IL-18 levels were similar to those found in healthy donors. Taken together, it can be speculated that the downregulation of IL-18 could be a consequence of leflunomide treatment. Moreover, to elucidate whether this involvement directly affects disease progression, a cross-sectional study before and after treatment is necessary.

Patients in DAS28 and CDAI, classified as moderate and severe, respectively, were divided into two groups, those who underwent leflunomide treatment and those who did not. IL-18 levels were lower in those treated with leflunomide, which led us to question how IL-18 influences the pathology of RA (data not shown).

In addition, IL-18 levels were lower in patients undergoing leflunomide treatment compared to the CG. Regarding the use of this medication and the activity of the disease (DAS28 and CDAI), it was observed that the patients who were not undergoing treatment exhibited higher levels of IL-18. In the Brazilian population, the IL-18 + 105 CC genotype and C allele were found to be associated with increased susceptibility to RA. These data indicated that the IL-18 gene polymorphism could be a possible genetic marker for susceptibility to RA [42].

Finally, we found that IL-16, IL-18, IL-31, and IL-32 are upregulated in Brazilian RA patients. Leflunomide has been shown as an active drug that potentially reduces IL-18 levels. It is still necessary to elucidate how this cytokine affects this disease and what the implications are for its downregulation. On the other hand, it is exceedingly difficult to find patients who take only one type of treatment; therefore, the study needs further corroboration to ensure leflunomide as the agent that causes IL-18 reduction.

## 5. Conclusions

We showed that IL-16, IL-18, IL-31, and IL-32 levels are upregulated in the serum of RA patients. More studies are needed to elucidate the role of IL-18 in RA, especially with the identification of reduced levels with leflunomide treatment. Understanding the role of leflunomide as a therapeutic agent and whether this is the most appropriate drug for RA patient treatment needs to be clarified.

## Figures and Tables

**Table 1:** Clinical and laboratory data for the RA and CG groups.

Data	RA	CG	*p*
Sample *n* (%)	140	40	0.0657
Female	130 (92.8%)	33 (82.5%)	
Male	10 (7.2%)	7 (17.5%)	

Age in years, mean ± SEM	54.0 (±12.0)	51.5 (±8.5)	0,0943
Race and ethnicity classifications			
Indigenous	1	0	*ns*
Multiracial	49	9	*ns*
Black or African American	60	22	*ns*
White	30	9	*ns*

Disease duration, years, mean ± SEM	10.0 (±7.8)		
+ rheumatoid factor (*n*%)	113 (80.7%)		
ESR mm/h, median, (range)	36 (2–125)		
Disease classification (*n*%)			
CDAI, mean ± SEM	18.5 (±13.0)		
Low disease activity (≤10)	45 (32%)		
Moderate and high disease activity (>10)	95 (68%)		
DAS28 mean ± SEM	4.5 (±1.5)		
Low disease activity (≤3.2)	31 (22.2%)		
Moderate and high disease activity (>3.2)	109 (77.8%)		

Treatment (*n*)			
None (naïve for treatment)	60		
Prednisone only	11		
Methotrexate only	12		
Leflunomide only	7		
More than one treatment	50		

Comorbidities (*n*)			
None	109		
Diabetes mellitus	9		
Obesity	6		
Osteoporosis	12		
Systemic arterial hypertension	24		

CG- control group; SEM- standard error of mean; ESR- erythrocyte sedimentation rate.

## Data Availability

Data are available on request from the authors.
